# Expression of myeloid differentiation factor 88 in neurons is not requisite for the induction of sickness behavior by interleukin-1β

**DOI:** 10.1186/1742-2094-9-229

**Published:** 2012-10-03

**Authors:** Theodore P Braun, Aaron J Grossberg, Biliana O Veleva-Rotse, Julia E Maxson, Marek Szumowski, Anthony P Barnes, Daniel L Marks

**Affiliations:** 1 Papé Family Pediatric Research Institute, Oregon Health and Science University, Portland, OR, 97239, USA; 2 MD/PhD Program, Oregon Health and Science University, Portland, OR, 97239, USA; 3 Knight Cancer Institute, Oregon Health and Science University, Portland, OR, 97239, USA; 4 Papé Family Pediatric Research Institute, Department of Pediatrics, Oregon Health and Science University, Mail Code L-481, 3181 SW Sam Jackson Park Rd., Portland, OR, 97239, USA

**Keywords:** Sickness behavior, MyD88, Cytokines, Inflammation, Hypothalamus, IL-1β, Cachexia, Lethargy

## Abstract

**Background:**

Animals respond to inflammation by suppressing normal high-energy activities, including feeding and locomotion, in favor of diverting resources to the immune response. The cytokine interleukin-1 beta (IL-1β) inhibits normal feeding and locomotor activity (LMA) via its actions in the central nervous system (CNS). Behavioral changes in response to IL-1β are mediated by myeloid differentiation factor 88 (MyD88) in non-hematopoietic cells. It is unknown whether IL-1β acts directly on neurons or requires transduction by non-neuronal cells.

**Methods:**

The Nestin-cre mouse was crossed with MyD88^lox^ mice to delete MyD88 from neurons and glia in the CNS (MyD88^ΔCNS^). These mice were compared to total body MyD88KO and wild type (WT) mice. Mice had cannulae stereotactically placed in the lateral ventricle and telemetry transponders implanted into the peritoneum. Mice were treated with either intracerebroventricular (i.c.v.) IL-1β (10 ng) or vehicle. Food intake, body weight and LMA were continuously monitored for 24 h after treatment. I.c.v. tumor necrosis factor (TNF), a MyD88-independent cytokine, was used to control for normal immune development. Peripheral inflammation was modeled using intraperitoneal lipopolysaccharide (LPS). Groups were compared using two-way ANOVA with Bonferroni post-test. Efficacy of recombination was evaluated using tdTomato reporter mice crossed with the Nestin-cre mouse. MyD88 deletion was confirmed by Western blot.

**Results:**

I.c.v. IL-1β treatment caused a significant reduction in feeding, body weight and LMA in WT mice. MyD88KO mice were protected from these changes in response to i.c.v. IL-1β despite having intact behavioral responses to TNF. Cre-mediated recombination was observed in neurons and astrocytes, but not microglia or endothelial cells. In contrast to MyD88KO mice, the behavioral responses of MyD88^ΔCNS^ mice to i.c.v. IL-1β or intraperitoneal (i.p.) LPS were indistinguishable from those of WT mice.

**Conclusion:**

Sickness behavior is mediated by MyD88 and is dependent on the activity of cytokines within the brain. Our results demonstrate that MyD88 is not required in neurons or astrocytes to induce this behavioral response to IL-1β or LPS. This suggests that a non-*Nestin* expressing cell population responds to IL-1β in the CNS and transduces the signal to neurons controlling feeding and activity.

## Background

Systemic inflammation elicits an evolutionarily conserved behavioral response, including anorexia, lethargy, anhedonia and hyperalgesia [1]. Collectively known as sickness behavior, this response is likely adaptive in the acute setting in that it reduces energy expenditure associated with foraging, but becomes pathological when present in a chronic state. Individuals suffering from chronic inflammatory illnesses, such as cancer, chronic renal failure, congestive heart failure and many others, exhibit decreased appetite and decreased energy to complete activities in the course of daily living [2]. Decreased nutritional intake consequent to decreased appetite contributes to ongoing weight loss in such individuals. Both weight loss and decreased activity not only negatively impact quality of life, but also are tightly correlated with mortality [3,4]. Despite this, there are currently no effective therapeutic interventions that alter the impact of the behavioral response to disease.

Sickness behavior is associated with increases in inflammatory cytokines, both circulating and in the central nervous system (CNS) [5]. Administration of inflammatory cytokines recapitulates the behavioral features of sickness, whereas cytokine antagonism has some efficacy in reversing these behaviors [6,7]. However, the mechanism by which inflammatory cytokines mediate behavioral changes remains incompletely described. Myeloid differentiation factor 88 (MyD88) is a signaling protein downstream from Toll-like receptors (TLRs) and the type I interleukin 1 receptor (IL-1RI). Sickness behavior in response to the inflammatory cytokine interleukin-1β (IL-1β) or the bacterial cell wall product LPS requires intact MyD88 signaling [8]. A series of bone marrow transplant experiments between WT and MyD88KO mice localized this requirement for MyD88 to a non-transplantable (that is, non-hematopoeitic) cell population [9]. Direct administration of IL-1β into the brain potently suppresses feeding and locomotor activity (LMA) indicating that cytokines act within the CNS to derive their behavioral effect [10]. Cytokine receptors are expressed on neurons known to regulate feeding and other behavioral responses, and neuron-specific disruption of these signaling pathways alters the behavioral responses to cytokine administration [11-13]. However, others provide evidence that cerebrovascular endothelial cells and perivascular macrophages are the targets of inflammatory cytokines in mediating sickness behavior [14-16].

To explore these distinct mechanisms of cytokine action and to establish the necessity of CNS MyD88 expression, we performed intracerebroventricular (i.c.v.) injections of IL-1β in MyD88 knockout mice (MyD88KO) and mice with a targeted deletion of MyD88 in the CNS only (MyD88^ΔCNS^). Our work here describes the feeding and locomotor responses to CNS inflammation, and the role of neuronal and astrocytic MyD88 in mediating this response.

## Methods

### Animals

Male C57BL/6 J wild type and MyD88KO (Stock# 009088) mice were purchased from The Jackson Labs (Bar Harbor, ME, USA). Nestin-cre mice (Stock# 003771) and MyD88 Flox mice (Stock# 008888) were obtained from The Jackson Labs, and crossed to obtained Nes-cre^+/#8722;^/MyD88 Flox^+/+^ mice. These mice were then crossed back to the original MyD88 Flox^+/+^ line to obtain MyD88^ΔCNS^ and MyD88^Flox^ littermate controls. Nestin-cre mice were also bred to mice harboring a Flox-Stop-Flox tdTomato behind a CAG promoter knocked into the ROSA locus (The Jackson Labs, Stock# 007908) or nuclear-localized green fluorescent protein/beta-galactosidase fusion protein (GNZ) (The Jackson Labs, Stock# 008606) to examine the cellular specificity of cre-mediated recombination. All mice were genotyped using standard protocols from The Jackson Labs. All animals were maintained on a normal 12:12 hr light/dark cycle and provided *ad libitum* access to water and food (Purina rodent diet 5001; Purina Mills, St. Louis, MO, USA). Mice were used for experiments at between 6 and 10 weeks of age. Experiments were conducted in accordance with the National Institutes of Health Guide for the Care and Use of Laboratory Animals, and approved by the Animal Care and Use Committee of Oregon Health and Science University.

### Intracerebroventricular injection

26-gauge lateral ventricle cannulas were placed (PlasticsOne, Roanoke, VA, USA) under isofluorane anesthesia, using a stereotactic alignment instrument (Kopf, Tujunga, CA, USA) at the following coordinates relative to bregma: -1.0 mm X, -0.5 mm Y and −2.25 mm Z. Ten ng mouse IL-1β or 500 ng mouse tumor necrosis factor (TNF, R&D, Minneapolis, MN, USA) injections were given in 1 μL total volume. IL-1β and TNF were dissolved in artificial cerebrospinal fluid (aCSF, 150 mM NaCl, 3 mM KCl, 1.4 mM CaCl_2_, 0.8 mM MgCl_2_, 1.0 mM NaPO_4_) with 0.1% endotoxin free BSA.

### LPS injection

LPS (Sigma, St. Louis, MO, USA) was dissolved at 62.5 μg/mL in 0.9% saline/0.5% endotoxin free BSA, and injected intraperitoneally at 4 μL/g body weight (250 μg/kg).

### Locomotor activity measurement

Voluntary home cage LMA was measured using implantable telemetric transponders (MiniMitter, Bend, OR, USA). Animals were anesthetized using 2% isoflurane, a small midline incision was made in the abdominal wall, and transponders were implanted adjacent to the abdominal aorta in the retroperitoneal space. Transponders were implanted during the lateral ventricle cannulation surgery. Mice were individually housed and allowed to acclimate for at least five days before temperature and net movement in *x-*, *y*- and *z*-axes was recorded in one minute intervals (Vital View, MiniMitter).

### Overnight feeding studies

Animals were transferred to clean cages and injected with i.c.v. IL-1β (10 ng), i.c.v. TNF (500 ng) or i.p. LPS (250 μg) 1 h prior to lights off. At 2, 6, 13, 24, 37 and 48 h after the onset of the dark cycle, food was weighed and returned to the cage. Body weight was recorded at 13, 24 and 48 h.

### Fast-refeed feeding studies

At lights out, food was removed from the cages and animals were fasted for 12 h. At the start of the light cycle, animals were injected i.c.v with IL-1β (10 ng) and food was returned to the cages 15 minutes later. Food intake was measured at 1, 2, 5, 8, 12 and 24 h after the return of food to the cages.

### Immunohistochemistry

For histology experiments, mice were deeply anesthetized using a ketamine cocktail and sacrificed by transcardial perfusion fixation with 15 mL ice cold 0.01 M PBS + heparin sodium (15,000 U/L) followed by 25 mL 4% paraformadehyde (PFA) in 0.01 M PBS. Brains were post-fixed in 4% PFA overnight at 4°C and cryoprotected in 20% sucrose for 24 h at 4°C before being stored at −80°C until used for immunohistochemistry (IHC). Dual-immunofluorescence histochemistry was performed as described below. Free-floating sections were cut at 30 μm from perfused brains using a sliding microtome (Leica SM2000R, Leica Microsystems, Bannockburn, IL, USA). Hypothalamic sections were collected from the division of the optic chiasm (bregma −1.0 mm) caudally through the mammillary bodies (bregma −3.0 mm). The sections were incubated for 1 h at room temperature in blocking reagent (5% normal donkey serum in 0.01 M PBS and 0.1% Triton X-100). After the initial blocking step, the sections were incubated in mouse anti-NeuN (1:1,000, Millipore, Billerica, MA, USA), rabbit anti-GFAP (1:500, DAKO, Carpinteria, CA, USA), rabbit anti-IbaI (1:500, DAKO), rat anti-CD31 (1:100, BD Pharmingen, Sparks, MD, USA) in blocking reagent for 72 h at 4°C, followed by incubation in donkey anti-rabbit Alexa 488 (1:500), donkey anti-mouse Alexa 488 or 647 (1:500), goat anti-rat Alexa 488 or 633 (1:500, Invitrogen) for 2 h at room temperature. Between each stage, the sections were washed thoroughly with 0.01 M PBS. Incubating the sections in the absence of primary antisera was used to ensure specificity of the secondary antibodies. Sections were mounted onto gelatin-coated slides, coverslipped using Vectashield mounting media (Vector Laboratories, Burlingame, CA, USA), and viewed on a Nikon A1 inverted laser-scanning confocal microscope (Nikon Instruments, Melville, NY, USA). Sections from Nestin-cre x GNZ animals were cut at 75 μm on a vibratome (Leica Microsystems, Buffalo Grove, IL, USA). Sections were incubated for 30 minutes in 98°C pH 6 sodium citrate buffer, then blocked as above. Sections were then incubated overnight at 4°C in 1:500 chicken anti-GFP antibody (Aves, Tigard, OR, USA). Sections were then washed as above, and incubated for 1 h in goat anti-chicken Alexa 568 and 647. Images were obtained as above, and both red and far red channels superimposed to generate higher anti-GFP signal.

### Neuronal cultures

Embryonic Day 14.5 (E14.5) timed pregnant female Nestin-cre/tdTomato mice were sacrificed by cervical dislocation and embryos were collected in cold complete HBSS. Heads were removed and cortices and hypothalami dissected and collected in cold complete HBSS. Tails were collected to confirm genotyping. Cortical and hypothalamic neurons were dissociated and were cultured for five days on Poly-D-Lysine-Laminin-coated glass coverslips in serum-free Gibco Neurobasal Medium containing Invitrogen penicillin-streptomycin and Invitrogen B-27 Supplement. Neurons were fixed in 4% paraformaldehyde in 1X PBS for 20 minutes at room temperature, then blocked and immunostained in a 1% BSA, 0.1% cold fish skin gelatin, 0.5% Triton-X 100, 10 mM TBS solution (blocking buffer). Neurons were immunostained overnight for MAP2 (1:1,000, Aves), Tau (1:10,000, Chemicon, Billerica, MA), NeuN (1:1,000, Chemicon) and βIII Tubulin (1:1,000, Chemicon) to confirm polarity and neuronal identity, respectively. Primary antibodies were applied overnight at 4°C and were washed off the next day 3x with 1XPBS. Hoechst and Alexa-Fluor secondary antibodies were used at 1:1,000 for one hour at room temperature in blocking buffer. Coverslips were mounted on slides and imaged on a Nikon A1 inverted laser-scanning confocal microscope. Neurons were scored for the presence of TdTomato using ImageJ software is open source free software from the National Institutes of Health. It is available at http://rsbweb.nih.gov/ij/download.html to determine efficiency of recombination.

### Western blot

Muscles were homogenized in cell lysis buffer (Cell Signaling, Danvers, MA, USA): 20 mM Tris pH 7.5, 150 mM NaCl, 1 mM EDTA, 1 mM EGTA 1% Triton X, 2.5 mM sodium pyrophosphate, 1 mM beta-glycerophosphate, 1 mM sodium orthovanadate, 1 ug/mL leupeptin, and supplemented with Complete protease inhibitors (Roche, Indianapolis, IN, USA), 1 mM PMSF (Sigma), and 5 μL/mL phosphatase inibitor cocktail II (Sigma). Samples were homogenized for 30 s using a Polytron homogenizer (Kinematica, Bohemia, NY, USA), then were sonicated 2 x 10s. Samples were then centrifuged at 13,000 RPM for 10 minutes at 4°C. For Western blots, 100 ug of total protein per lane was run on Criterion 4 to 15% gradient polyacrylamide gels (Biorad, Hercules, CA, USA), and transferred to Immobilon PVDF membranes (Millipore. Proteins were detected using a Lumi-Imager (Roche) and SuperSignal West Pico Chemiluminescent Substrate (Thermo, Rockford, IL, USA). The following antibodies were used: rabbit anti-MyD88 (Cell Signaling) and an anti-rabbit HRP conjugate (Promega, Madison, WI, USA).

## Results

**Figure 1:**
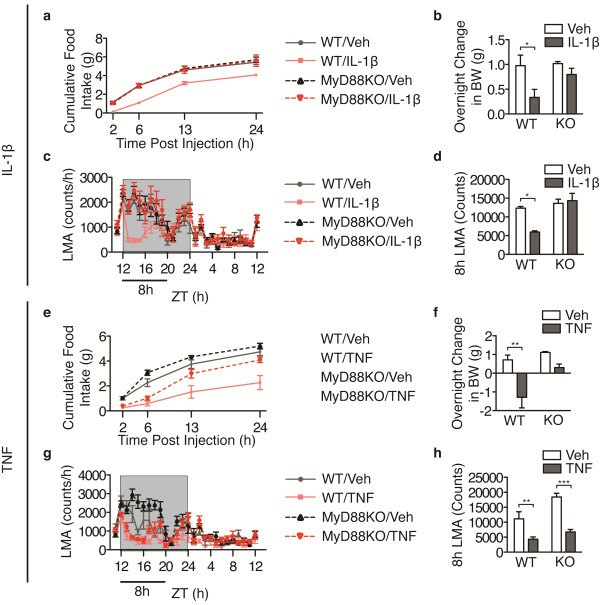
**MyD88 is required for sickness behavior in response to central IL-1β injection.** WT and MyD88KO mice received i.c.v. injections of IL-1β (10 ng) or TNF (500 ng) 1 h before the onset of the dark cycle (n = 5 to 6/group). (**a**, **e**) Cumulative food intake. (**b**, **f**) Overnight change in body weight. (**c**, **g**) Hourly locomotor activity. (**d**, **h**) Cumulative locomotor activity over 8 h post injection. All data are represented as mean ± s.e.m. In some cases, error bars are too small to see. KO, MyD88KO; ZT, Zeitgeber Time. ***, *P* <0.001; **, *P* <0.01; *, *P* <0.05 as measured by two-way ANOVA with Bonferroni post-test.

MyD88 is requisite for sickness behavior in response to centrally administered cytokine MyD88 is essential for conveying the anorectic signal in response to peripheral LPS or IL-1β treatment. It has been proposed that fast IL-1β signaling in anterior hypothalamic neurons occurs in a phosphatidylinositol 3-kinase (PI3K)-dependent, MyD88-independent mechanism [17]. To examine whether MyD88 is requisite for the behavioral response to cytokines administered directly into the brain, we investigated the response of MyD88KO mice to i.c.v injection of IL-1β. Injections of IL-1β (10 ng) or vehicle (veh) were performed one hour before lights off at Zeitgeber time (ZT) 11 to 12. Although WT mice showed a significant suppression of dark-phase food intake in response to IL-1β (Figure 1a), feeding in MyD88KO mice was unperturbed. WT mice lost body weight in response to IL-1β treatment, whereas MyD88 mice were undistinguishable from veh-treated controls (Figure 1b). WT mice treated with IL-1β showed significant suppression of home cage LMA for the first 8 h of the dark cycle, whereas MyD88KO mice treated with IL-1β exhibited normal LMA (Figure 1c, d). These data demonstrate that MyD88 is required for sickness behavior in response to IL-1β applied into the lateral ventricle. 

To validate that MyD88KO mice are capable of displaying sickness behavior in response to a MyD88-independent inflammatory stimulus, we performed i.c.v. injections of the MyD88 independent cytokine TNF (500 ng) in MyD88KO mice. As TNF is a far less potent inducer of sickness behavior than IL-1β, and the purpose of this experiment was not to model physiologic inflammation, but to rule out developmental limitations, a relatively high dose of the cytokine was used. Anorexia, weight loss and decreased LMA were all present in MyD88KO mice treated with TNF during the first six hours after treatment (Figure 1e-h). During the second half of the dark phase, TNF-treated MyD88KO mice consumed more food than veh-treated controls such that no differences in cumulative food intake were apparent 13 h after treatment. Thus, MyD88KO mice display normal sickness behavior at early time points in response to an inflammatory challenge that does not require MyD88. The limited duration of this effect demonstrates that MyD88 is important for the propagation of sickness behavior beyond the acute period irrespective of the inciting inflammatory stimulus.

### Nes-cre mice exhibit cre-mediated recombination in neurons

**Figure 2:**
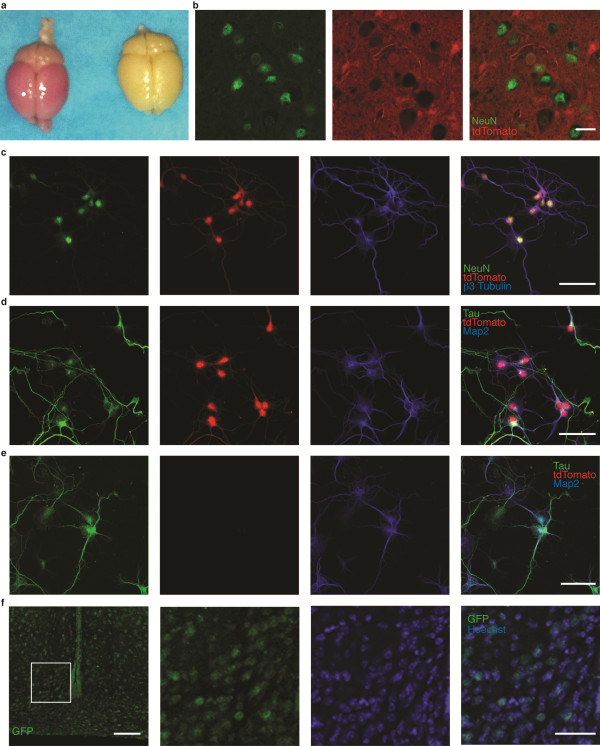
**Nestin-cre drives recombination in neurons.** (**a**) Gross images of tdTomato x Nes cre mouse brains showing diffuse expression of the tdTomato protein throughout the central nervous system. Brains from Nes-cre/tdTomato reporter mice were immunostained with antibodies against NeuN labeling neuronal nuclei. Images seen in (**b**) show high magnification of brains from Nes-cre/tdTomato reporter mice immunostained with antibodies against NeuN to examine co-expression. High power photomicrographs showing cultured cortical neurons from E14.5 Nes-cre/tdTomato mice immunostained with antibodies against neuron-specific proteins NeuN and β3 tubulin (**c**) or Tau and Map2 (**d**). Cultured cortical neurons from E14.5 Nes-cre^−/−^/tdTomato negative control mice stained for Tau and Map2 using the same microscope settings as in (**c**). Tissue sections from Nes-cre/GNZ mouse hypothalami demonstrating dense nuclear GFP expression (**f**). Representative images in (f) of the ARC at −1.20 to −1.70 mm relative to bregma. Scale bars, (**b**) 10 μm, (**c**, **d**, **e**) 50 μm, (**f**) 100 μm-low power; 50 μm-high power.

To explore the identity of the cell population in which MyD88 is requisite for the behavioral response to inflammation, we generated mice lacking MyD88 exclusively in the central nervous system (MyD88^ΔCNS^). Mice expressing cre recombinase under the control of the rat *nestin* promoter were crossed with mice harboring an allele of MyD88 where exon 3 of the gene is flanked by LoxP sites to generate CNS specific deletion of MyD88. These mice display no overt phenotype until challenged with a high fat diet, despite appropriate recombination [18]. While the Nestin-cre mouse has been utilized extensively, reports vary as to the precise identity of cells in the CNS exhibiting recombinase activity. To clarify this issue, we crossed the Nestin-cre mouse to an inducible reporter in which the tdTomato fluorescent protein is expressed in cells that have expressed cre recombinase at any point during their development. Upon dissection following perfusion fixation, the brains were grossly red compared to WT (Figure 2a). Although tdTomato fluorescence is widely visualized throughout the coronal brain sections, this staining is specific, as evidenced by sharply demarcated nuclear clearing. In the hypothalamus, high power confocal images demonstrate nuclear clearing of the cytosolic tdTomato and staining of a subnuclear compartment by the neuronal marker NeuN, indicative of cre-mediated recombination in neurons (Figure 2b). Thus, the tdTomato protein appears to be only cytosolic in the vast majority of neurons, which is consistent with multiple reports examining the neuronal expression of genetically-encoded fluorescent proteins that lack a nuclear localization signal [19]. 

To further confirm recombination in neurons, we isolated and cultured hypothalamic and cortical neurons from Nestin-cre/tdTomato reporter mice and costained them with antibodies against neuronal markers NeuN, Map2, Tau and β3 Tubulin. We did not observe any cultured cells with neuronal phenotype that did not express tdTomato fluorescence (Figures 2c, d). As a control, neurons from tdTomato reporter mice lacking cre-7expression were harvested, stained and visualized using identical microscope settings. We observed no red fluorescence in any control mice, verifying the specificity of the Nestin-cre recombination (Figure 2e). Because tdTomato is only expressed in the cytoplasm, we wanted to verify that the diffuse tdTomato fluorescence accurately represented pan-neuronal recombination. To do so we crossed the Nes-cre mouse with the GNZ reporter mouse, in which nuclear-localized GFP is conditionally expressed in cells expressing cre-recombinase. We observed densely labeled nuclei in all brain regions, including the hypothalamus, with absence of staining in the cytoplasm, consistent with pan-neuronal recombination (Figure 2f).

### Nes-cre mice exhibit cre-mediated recombination in astrocytes, but not microglia or cerebrovascular endothelial cells

**Figure 3:**
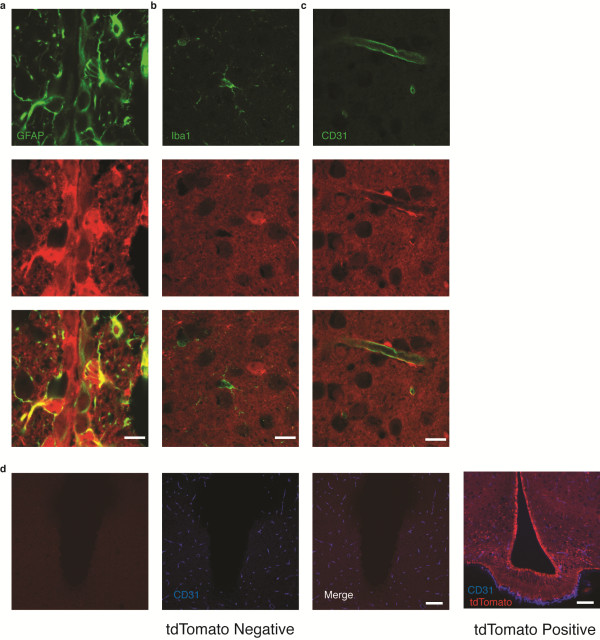
**Nestin-cre drives recombination in astrocytes but not endothelium or microglia.** Brains from Nes-cre tdTomato reporter mice were immunostained with antibodies against (**a**) GFAP marking astrocytes, (**b**) Iba1 labeling microglia or (**c**) CD31 labeling endothelium. (**d**) tdTomato negative and tdTomato positive sections doubled labeled for CD31. Scale bar represents 10 μm for a, b, c and 100 μm for d.

We also examined the expression of tdTomato in other CNS cell types. GFAP positive astrocytes clearly express tdTomato consistent with recombination in this cell type (Figure 3a). However, cre recombinase activity is not present in microglia (marked by Iba1) or endothelium (marked by CD31), as demonstrated by a lack of tdTomato expression in these cell types (Figure 3b, c). To confirm the specificity of the tdTomato signal, hypothalamic sections from tdTomato negative and positive animals were examined using the same microscope settings, with significantly increased signal evident in the red channel for tdTomato positive animals (Figure 3d).

### Brain MyD88 expression is significantly reduced in MyD88^ΔCNS^ mice

**Figure 4:**
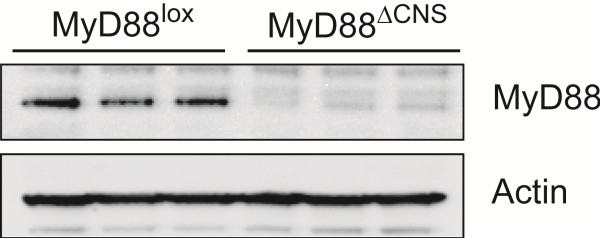
**MyD88 expression is reduced in brains from MyD88**^**ΔCNS**^**mice.** Western blots were performed on equal amounts of proteins from homogenized brains from MyD88^ΔCNS^ or MyD88^Lox^ control mice using antibodies against MyD88. Three replicate results from one experiment are shown. Replicates were immunostained for actin as a loading control.

To verify that MyD88 was deleted from the brains of MyD88^ΔCNS^ animals, we examined MyD88 protein expression from MyD88^ΔCNS^ and MyD88^lox^ control mice. Western blot analysis demonstrates a marked reduction in MyD88 protein expression in brains from MyD88^ΔCNS^ mice compared to control animals (Figure 4). A very faint band can be seen in the MyD88^ΔCNS^ mice, consistent with residual expression in non-Nestin expressing microglia and cerebrovascular endothelial cells.

### Deletion of MyD88 from neurons and astrocytes does not alter sickness behavior in response to IL-1Î² or LPS

**Figure 5:**
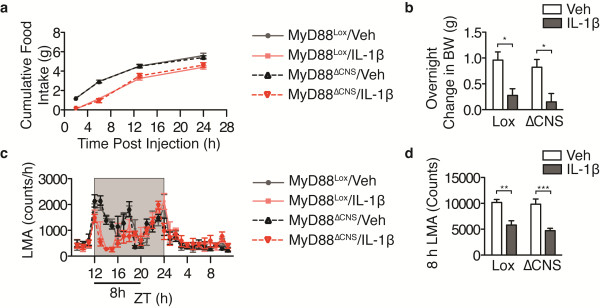
**MyD88 expression in the CNS is not required for IL-1β-induced sickness behavior.** WT and MyD88^ΔCNS^ mice received i.c.v. injections of IL-1β (10 ng) 1 h before the onset of the dark cycle (n = 4 to 9/group). (**a**) Cumulative food intake. (**b**) Overnight change in BW. (**c**) Hourly locomotor activity. (**d**) Cumulative locomotor activity over 8 h post injection. All data are represented as the mean ± s.e.m. In some cases error bars are too small to see. Lox, MyD88^Lox/lox^; ΔCNS, MyD88^ΔCNS^; ZT, Zeitgeber Time. ***, *P* <0.001; **, *P* <0.01; *, *P* <0.05 as measured by two-way ANOVA with Bonferroni post-test.

**Figure 6:**
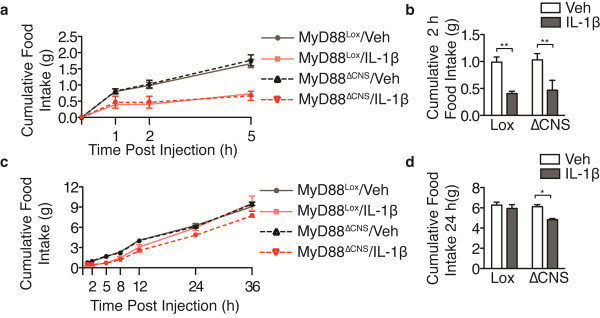
**MyD88 expression in the CNS is not required for IL-1β-induced suppression of refeeding.** WT and MyD88^ΔCNS^ mice were fasted overnight for 12 h and then received i.c.v. injections of IL-1β (10 ng) 15 minutes prior to food being returned to the cages (n = 4 to 5/group). (**a**) Cumulative food intake 0 to 5 h. (**b**) Cumulative food intake at 2 h. (**c**) Cumulative food intake 0 to 36 h. (**d**) Cumulative food intake at 24 h. All data are represented as the mean ± s.e.m. ΔCNS, MyD88^ΔCNS^. ** ; *P* <0.01, * ; *P* <0.05 as measured by two-way ANOVA with Bonferroni post-test.

We assessed the behavioral response of MyD88^ΔCNS^ mice to IL-1β administered prior to the onset of the dark cycle (ZT 11–12). MyD88^ΔCNS^ mice displayed normal suppression of overnight feeding and body weight in response to i.c.v. IL-1β (Figure 5a, b). Spontaneous home cage activity was suppressed equally in MyD88^ΔCNS^ and MyD88^lox^ mice treated with i.c.v. IL-1β (Figure 5c, d). It is possible that the neuronal response to i.c.v. IL-1β is transient, requiring inflammatory amplification by a population of cells not expressing cre, such as microglia or endothelium, to sustain the anorectic response. Were this the case, attenuation of the behavioral response to i.c.v. IL-1β in the MyD88^ΔCNS^ mouse would only be observable at early time-points prior to amplification of this signal into a polycytokine response, which would circumvent the block in neuronal MyD88 signaling. To evaluate this possibility, we repeated these experiments in mice fasted for 12 h prior to i.c.v. IL-1β treatment. Fasting increases the relative fraction of food intake occurring within the first two hours, enhancing the sensitivity of the experiment to acute feeding differences. Again, IL-1β was equally effective at reducing food intake in both MyD88^ΔCNS^ and MyD88^lox^ mice at all time points (Figure 6a-d). Collectively, these data demonstrate that MyD88 expression in neurons and astrocytes is dispensable for IL-1β-induced sickness behavior.

### MyD88 is not required in neurons or astrocytes for LPS-induced sickness behavior

**Figure 7:**
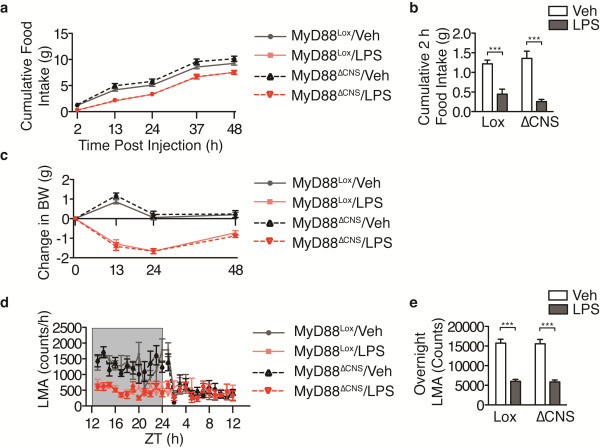
**MyD88 expression in the CNS is not required for LPS-induced sickness behavior.** WT and MyD88^ΔCNS^ mice received i.p. injections of LPS (250 μg/kg) 1 h before the onset of the dark cycle (n = 4 to 9/group). (**a**) Cumulative food intake. (**b**) Cumulative food intake at 2 h post injection. (**c**) Change in body weight after LPS injection. (**d**) Hourly locomotor activity. (**e**) Cumulative locomotor activity during the entire night cycle. All data are represented as the mean ± s.e.m. In some cases error bars are too small to see. Lox, MyD88^Lox/lox^; ΔCNS, MyD88^ΔCNS^; ZT, Zeitgeber Time. *** , *P* <0.001 as measured by two-way ANOVA with Bonferroni post-test.

LPS derives its anorectic effects through enhanced IL-1β expression within the brain [7]. Although inflammatory signaling pathways downstream of TLR4 can be activated independent of MyD88, MyD88KO mice fail to mount a cytokine response to treatment with the TLR4 ligand, LPS [20]. Furthermore, IL-1β immunoreactivity has been observed in both astrocytes and neurons [21,22]. To evaluate whether MyD88 is necessary in these cell types for the behavioral response to LPS, we treated MyD88^ΔCNS^ mice with peripheral LPS injections. One hour prior to the onset of the dark cycle, MyD88^ΔCNS^ mice were injected i.p. with LPS (250 μg/kg) or veh*.* Overnight and 2 h food intake was reduced in both MyD88^Lox^ and MyD88^ΔCNS^ mice (Figure 7a, b). LPS treatment resulted in a significant loss of body weight, without evident differences between genotypes (Figure 7c). Home cage activity was suppressed by LPS administration for the entire duration of the dark cycle (Figure 7d, e). Therefore, expression of MyD88 in neurons or astrocytes is not required for LPS-induced sickness behavior. 

## Discussion

Sickness behavior is an integral component of the adaptive metabolic response to infection. In chronically ill patients this response becomes maladaptive, significantly worsening quality of life and potentially contributing to mortality. Despite numerous mechanistic inroads into the pathogenesis of sickness behavior, the precise cell type upon which IL-1β acts to produce sickness behavior has remained elusive. In this work, we demonstrate a requirement for MyD88 in transducing IL-1β within the brain into sickness behavior. Although MyD88 expression in neurons has been suggested to play a critical role in the behavioral response to inflammation, we find that IL-1β induced anorexia and lethargy is not contingent upon MyD88-expression in both neurons and astrocytes.

The receptor for IL-1β is expressed in multiple locations within the CNS, many of which are known to regulate food intake. In particular, IL-1R1) is expressed in the arcuate nucleus of the hypothalamus (ARC) on anorectic proopiomelanocortin (POMC) and orexigenic Agouti Related Peptide (AgRP) neurons. IL-1β increases the firing of POMC neurons, and increases the release of alpha-melanocyte stimulating hormone (α-MSH), the natural ligand of the type-4 melanocortin receptor (MC4R), from hypothalamic explants [12]. IL-1β simultaneously decreases the release of AgRP, the endogenous antagonist of the MC4R [13]. Consistent with this, mice lacking the MC4R resist IL-1β induced anorexia, demonstrating that alterations in melanocortin signaling underlie changes in feeding in response to IL-1β [23].

Despite clear recombination in the ARC in the tdTomato reporter mouse, MyD88^ΔCNS^ mice respond normally to IL-1β. There are several possible explanations to resolve these apparently conflicting results. Cre mediated recombination of the MyD88 allele may be incomplete, with a sufficient number of neurons remaining responsive to IL-1β in the MyD88^ΔCNS^ mouse to engender a normal anorectic response. If the results of our tissue and cell culture IHC are representative, less than 1% of neurons are sufficient to mediate these behaviors. Alternatively, it is possible that IL-1β produces anorexia in an IL-1RI mediated, MyD88-independent manner. There is some precedence for this, as it has been proposed that IL-1β signals via a PI3K dependent mechanism in neurons, similar to the mechanism by which leptin mediates electrophysiologic changes in neurons [17]. A third possibility is that cytokine signaling has an obligate amplification step in a cell type that does not undergo recombination in the MyD88^ΔCNS^ mouse, leading to the paracrine release of a second messenger that then conveys the anorectic signal to neurons.

The identity of this second messenger remains in question. Some propose that prostaglandins convey the inflammatory signal to neurons [15,16,24]. Whereas this is clearly the case for fever [25], cyclooxygenase inhibition fails to alter the changes in volitional activity observed after i.p. LPS injection [10], and decreases, but does not block, the anorectic response to peripheral and central inflammatory challenges [11,26,27]. Therefore, while prostaglandins mediate some of the behavioral response to systemic inflammation, other second messengers must also relay inflammatory signals to the neurons regulating behavior.

IL-1β itself may be the ultimate mediator of inflammation-induced anorexia. Previous work by Konsman *et al.* demonstrates that i.c.v. administration of IL-1β to rats diffuses throughout the parenchyma, eliciting neuronal activation [28]. These cellular responses may be secondary to direct activation of the IL-1RI on neurons by IL-1β that has diffused from the ventricles. It is also possible that IL-1β, at the doses given in the present study, does not reach sufficient concentration after i.c.v. injection to engage the IL-1RI on feeding center neurons, yet is amplified locally by a non-neuronal cell. There is some experimental evidence to support this mechanism. First, i.c.v. IL-1β exhibits preferential perivascular diffusion, suggesting a role for perivascular or endothelial amplification in its acute effects [28]. Second, IL-1β mRNA increases specifically in the parenchyma of the ARC following i.c.v. IL-1β or i.p. LPS administration [29]. When IL-1 receptor antagonist (IL-1Ra) is administered at a high dose during peripheral endotoxemia, anorexia is prevented, demonstrating that endogenous brain IL-1 signaling is integral to the anorectic response [7]. Endogenous IL-1 signaling also plays a role in mediating protein catabolism, as i.c.v. infusion of IL-1Ra attenuates the loss of muscle mass in experimental sepsis models [30]. However, in order for this mechanism to be consistent with the presented data, the induction of hypothalamic IL-1β would have to be dependent on MyD88, yet its ultimate action on neurons is independent from MyD88. As evidence exists from other cell types that cytokine production is dependent on MyD88 [31], and IL-1β signaling on neurons is MyD88 independent [17], this remains a plausible explanation for the data presented here.

Logical targets for IL-1β signaling in the context of these findings are cerebrovascular endothelial cells. Endothelial knockdown of the IL-1RI blocks the suppression of home cage LMA after i.c.v. IL-1β injection and prevents the activation of neurons in the paraventricular nucleus (PVN) of the hypothalamus, which is associated with hypothalamic-pituitary-adrenal, sympathetic nervous system and anorectic responses to inflammation [15]. Furthermore, brain endothelial specific knockout of transforming growth factor β-activated kinase attenuates the depression in LMA seen after IL-1β administration [16]. Perivascular macrophages have also been implicated as key intermediaries in the central response to inflammation. Selective depletion of perivascular macrophages decreases the activation PVN neurons in response to peripheral IL-1β but does not alter the depression of LMA [14]. Neither endothelium nor microglia show cre-mediated recombination in the Nestin-cre mice. Therefore, endothelium and perivascular macrophages likely represent a component of the cell population in which MyD88 is required for sickness behavior.

## Conclusions

In summary, our results demonstrate that MyD88 is not required in neurons or astrocytes to induce the behavioral response to i.c.v. IL-1β. This suggests that a non-*nestin* expressing cell population in the CNS responds to IL-1β and transduces the signal to neurons controlling feeding and activity. We posit that cerebrovascular cells or perivascular macrophages/microglia are the most likely targets for IL-1β signaling in the brain, implying that systemic therapy addressing anorexia and fatigue need not penetrate the blood brain barrier.

## Abbreviations

α-MSH: Alpha-melanocyte stimulating hormone; aCSF: Artificial cerebrospinal fluid; AgRP: Agouti-related peptide; ARC: Arcuate nucleus of the hypothalamus; BSA: Bovine serum albumin; CD31: Cluster of differentiation 31; CNS: Central nervous system; GFAP: Glial fibrillary acidic protein; Iba1: Ionized calcium binding adaptor molecule 1; i.c.v.: Intracerebroventricular; IHC: Immunohistochemistry; IL-1β: Interleukin-1 beta; i.p.: Intraperitoneal; IL-1Ra: IL-1 receptor antagonist; IL-1RI: Interleukin-1 receptor I; LMA: Locomotor activity; LPS: Lipopolysaccaride; Map2: Microtubule associated protein-2; MC4R: Type-4 melanocortin receptor; MyD88: Myeloid differentiation factor 88; MyD88^ΔCNS^: CNS-specific MyD88 knockout mouse; MyD88KO: Myeloid differentiation factor 88 knockout mouse; NeuN: Neuronal nuclear antigen; PI3K: Phosphatidylinositol 3-kinase; PBS: Phosphate-buffered saline; PFA: Paraformaldehyde; PMSF: Phenylmethanesulfonylfluoride; POMC: Proopiomelanocortin; PVN: Paraventricular nucleus of the hypothalamus; TLR: Toll-like receptor; TNF: Tumor necrosis factor; veh: Vehicle; WT: Wild type; ZT: Zeitgeber time.

## Competing interests

The authors declare that they have no competing interests.

## Authors’ contributions

TPB and AJG designed the studies; carried out the surgeries, physiology studies, immunohistochemical studies, and data analysis; and wrote the manuscript. BOV conducted the neuronal cell culture and GNZ mouse studies. JEM performed the Western blotting and analysis. MS managed the mouse colony and contributed experimentally. APB contributed to the design and analysis of the study. DLM contributed to the design and analysis of the study and wrote the manuscript. All authors read and approved the final manuscript.
